# Aggregating, Tagging and Integrating Biodiversity Research

**DOI:** 10.1371/journal.pone.0019491

**Published:** 2011-08-01

**Authors:** David P. Mindell, Brian L. Fisher, Peter Roopnarine, Jonathan Eisen, Georgina M. Mace, Roderic D. M. Page, Richard L. Pyle

**Affiliations:** 1 California Academy of Sciences, San Francisco, California, United States of America; 2 Department of Medical Microbiology, Section of Evolution and Ecology, University of California Davis, Davis, California, United States of America; 3 Division of Biology, Imperial College London, London, United Kingdom; 4 University of Glasgow, Institute of Biodiversity, Animal Health and Comparative Medicine, Glasgow, United Kingdom; 5 Department of Natural Sciences, Bishop Museum, Honolulu, Hawaii, United States of America; University of Bristol, United Kingdom

Scientists are amassing details about the scope and status of life's variation at an accelerating rate. This aids our understanding of species' distributions and their interactions over space and time. If we are to address the consequences of global environmental change for life's future, however, biodiversity data must be aggregated, integrated and synthesized to a much greater degree than they are at present. Here, we call attention to a new community resource and tool which provides a step in the right direction.

*PLoS* has launched a Biodiversity Hub < http://hubs.plos.org/web/biodiversity/ > aiming to accelerate the discovery, dissemination and integration of biodiversity studies. Biodiversity is broadly construed as life's variation, including the richness, relationships, functions and distribution of genes, species, communities, and ecosystems across terrestrial, marine and freshwater realms. Biodiversity studies often integrate evolution, ecology and conservation science to better understand and conserve life's variation.

The Biodiversity Hub provides three general services. First, the Hub builds on the idea of open access publication by aggregating selected open access journal articles focused on biodiversity science. Second, the Hub adds value to previously published content. Initially, that value will involve links to images, distribution maps, publications and data about species featured in the articles. Eventually, we hope the Hub will integrate the semantic mark-up of taxonomic and other biodiversity science elements within open-access papers. Thus, papers aggregated by the Hub can include digital images, maps, and data that are not shown in the original publication, and semantically tagged elements (e.g., species names) that can make such information much easier to find and synthesize. We are hopeful that this will improve professional and public access to biodiversity data, allowing broader use of the information in research and raising public awareness of biodiversity issues. Third, the Hub provides a community forum for interaction around specific content. Commentary and links to research resources and community projects can attract users and broaden support for biodiversity initiatives. The Hub can bring biodiversity publications to life.

Over the past decade, there has been considerable progress in synthesizing and digitizing biodiversity-related assets. Resource assets include aggregated specimen data: *GBIF* (<http://www.gbif.org/>), *The Paleobiology Database* (<http://www.paleodb.org>); interoperability among datasets and databases: *GEO BON* (<http://www.earthobservations.org/geobon.shtml>), *Global Names Architecture* (<http://globalnames.org>); taxonomic literature: *BHL* (<http://www.biodiversitylibrary.org/>); taxonomic names: *Zoobank* (<http://www.zoobank.org/>), *IPNI (* http://www.ipni.org/ *)*, *Catalogue of Life* (<http://www.catalogueoflife.org>), *Index Fungorum* (<http://www.indexfungorum.org/>); molecular sequence data: *GenBank* (<http://www.ncbi.nlm.nih.gov/Genbank/>), *Barcode of Life* (<http://www.dnabarcodes.org/>), *Greengenes* (<http://greengenes.lbl.gov>); images: *MorphBank* (<http://www.morphbank.net/>), *ARKive* (<http://arkive.org/>); phylogenetic relationships: *Tree of Life* (<http://tolweb.org/tree/>), *TreeBASE* (<http://www.treebase.org>); natural history: *Encyclopedia of Life* (<http://www.eol.org/>); conservation status of species: *IUCN Red List* (<http://www.iucnredlist.org/>), *WWF Wildfinder* (<http://gis.wwfus.org/wildfinder/>); and ecological and evolutionary datasets (<http://datadryad.org/>).

A lack of integration of these and similar assets, with each other and with the publication process, limits a realization of their full potential. Integrating biodiversity resources depends on linking datasets to analyses, using shared global identifiers, and deploying services that link those identifiers [1], [2]. These steps and others can enhance access and stability over time for the units of analyses, such as individual museum specimens, taxon names, geographic locations, and molecular sequences. Integration of such basic biodiversity elements in publications can result in greater credit going to the investigators and providers of these scientific data. The current lack of attribution for the continuing use of hard-won, primary biodiversity data (e.g., species descriptions, character data sets for phylogenetic analyses, long-term field study observations, curation of biodiversity databases) leads universities and funding agencies to underestimate the value of biodiversity-related disciplines.

The integration of assets and open access to them can help to change this situation [3]. For a summary of principles of knowledge-sharing for the Conservation Commons and biodiversity data, see http://www.conservationcommons.net/. The *PLoS* Biodiversity Hub offers a chance to address some of these shortcomings and opportunities in the publication process and subsequent use of the literature, while simultaneously enhancing *PLoS*'s overall mission to disseminate, synthesize, and connect scientific knowledge (see [4], [5]).

The most basic challenges for biodiversity scientists are the more thorough description and comprehension of life's variation. Providing names and phylogenetic classifications, where possible, is an integral part of this task. Current progress in the cataloging of species richness can be summarized as follows. Roughly 1.7 million species of multi-cellular organisms have been described and recognized, including about 62,000 vertebrates, 1,300,000 invertebrates, 321,000 plants and 51,500 fungi and brown algae [6]. Diversity and numbers of single-celled life forms including protists, Bacteria, Archaea, and viruses are less extensively described and cannot in many or most cases be tallied in the same way due to asexual reproduction, frequent lateral gene transfer and differing species concepts. The total number of extant species on Earth is not known, though recent estimates range from 5 to 30 million [7], with the most plausible estimates lying near the lower end of the range. Thus, we may have described between 5.7% to 34% of species with a strong bias to the macrofauna and flora. Taxonomic discovery is not complete for any of the major groups; however, the gaps in our knowledge are particularly acute for insects, fungi, algae and unknown legions of single-celled organisms. Efforts to bridge these gaps should be a priority, as these poorly known groups are important components of healthy, functional ecosystems, and play key roles in nutrient cycling, decomposition, pollination, symbioses, and soil fertility, and as primary producers and consumers. Biases exist also on the basis of habitat and environment, with deep ocean and deep earth environments being poorly known relative to more accessible areas. Similarly, the diversity of small life forms living in or on other species is greatly under-sampled. Documenting the numbers of species and their biotic interactions are fundamental to understanding the complexity, robustness and functioning of the ecosystems comprising those species.

Museum specimen and species databases useful for macroorganism research are growing and becoming more widely accessible. Despite the emergence of other sources of species information in recent decades, museum data still represent an unparalleled source of broad-based historical information on species [8]. Increasingly, museum databases incorporate digital images, GIS data, maps, mensural data, and links to species descriptions and other natural history references. Comparative molecular data sets from diverse species also continue to grow, and are providing our best understanding of microorganism diversity and evolution. An estimate from the *Genomes OnLine Database* (GOLD; <http://www.genomesonline.org/>) in March 2011 of the number of genome projects completed and in progress includes 5,843 Bacteria, 2,003 Eukarya species and 210 Archaea. In many cases, annotations of gene and character homology relationships and the structure and function of genes and proteins are improving, though much work is needed in this area.

Although our basic knowledge of biodiversity is increasing, analyses based on the best available long-term data sets indicate that the high rates of decline in diversity for many organismal groups continue undiminished [9]. Estimates of the extinction risks for animals, plants and fungi are accelerating while negative pressures exerted on those species are increasing, including climate change impacts, habitat destruction, unsustainable exploitation, spread of invasive species and ongoing human consumption and competition for natural resources.

The reality of species loss and changing distributions resulting from human activities lends urgency to the study of connections between the health of biological diversity, the health of ecosystems and ultimately the health of human populations. The connections linking biological diversity and human well-being are traditionally discussed in terms of ecosystems services. These include the many benefits that people derive from nature, such as potable water, productive soils and nutritious food; regulation of climate and infectious diseases; provision of medicinal and genetic resources; and impacts on quality of life [10], [11]. Adding an evolutionary perspective to these linkages has been proposed recently with use of the term “evosystem services” [12]. This explicitly recognizes the value of evolutionary processes in generating and maintaining biodiversity and its many valuable products.

The Biodiversity Hub is a work in progress. The site is maintained by *PLoS* staff working with Hub curators and a steering committee. Current technical limitations include being restricted to open access articles indexed by *PubMed Central* and not having mechanisms for quickly adding articles or for semantic tagging of species names. At present, the articles aggregated by the Biodiversity Hub are not sorted in any way, and we think that restructuring the site to include taxonomic and conceptual or methodological section headings could provide an intuitive, meaningful entry point to the large set of publications. There is a need for development of efficient web tools for aggregating and curating articles, and for tagging species names and linking them to relevant sources. The latter tasks are particularly challenging, given the many gaps in our knowledge of species diversity and the incomplete nature of species names databases. Ideally, the Hub could be maintained and curated by the community of biodiversity scientists without requiring extensive resources from *PLoS*.

At present, the site includes a series of previously published, open access biodiversity articles selected by Hub curators. This includes several articles in which species names have been semantically tagged by hand, to illustrate the potential benefits of semantic tagging and of linking automatically to various databases, maps and unique global identifiers. Interested readers should follow links to these publications on the Biodiversity Hub and explore the tagged species names: Fisher and Smith 2008 <hubs.plos.org/web/biodiversity/article/10.1371/journal.pone.0001787> [13], Walston et al. 2010 <hubs.plos.org/web/biodiversity/article/10.1371/journal.pbio.1000485> [14], and Johnson et al. 2006 <hubs.plos.org/web/biodiversity/article/10.1186/1471-2148-6-65> [15].

This attempt at automated synthesis for some biodiversity data is far from comprehensive, and shows how much work remains to be done. However, extending such efforts to integrate biodiversity knowledge will enhance information exchange, raise awareness of the great value of primary biodiversity data, improve attribution of credit for species discovery and taxonomy, and facilitate syntheses of the causes and consequences of global biodiversity change. These may, in turn, lead to better understanding of the ways in which humans can manage and adapt to a changing world.
